# LINC01094 promotes gastric cancer through dual targeting of CDKN1A by directly binding RBMS2 and HDAC1

**DOI:** 10.1186/s13062-024-00582-y

**Published:** 2024-12-25

**Authors:** Xinyi Zhou, Cheng Gu, Linmei Xiao, Li Hu, Guanhua Chen, Fei Zuo, Hongan Shao, Bojian Fei

**Affiliations:** 1 Department of Gastrointestinal Surgery, Affiliated Hospital of Jiangnan University, 1000 Hefeng Road, Wuxi, 214062 Jiangsu Province China; 2 Department of Joint Surgery, The First Affiliated Hospital, Sun Yat-Sen University, Guangzhou, 510080 China; 3 Department of Liver Disease, Wuxi No.5 People’s Hospital Affiliated to Jiangnan University, Wuxi, 214000 Jiangsu Province China; 4 Department of Gastrointestinal Surgery, The First Affiliated Hospital of Ningbo University, Ningbo, 315000 Zhejiang Province China; 5 Department of General Surgery, The First Affiliated Hospital of Nanjing Medical University, Nanjing, 210000 Jiangsu Province China; 6 Department of Radiation Oncology, Nanjing Jinling Hospital, Affiliated Hospital of Medical School, Nanjing University, Nanjing, 210000 Jiangsu Province China; 7 Nanjing BenQ Medical Center, The Affiliated BenQ Hospital of Nanjing Medical University, Nanjing, 210019 Jiangsu Province China; 8 Department of Thoracic Surgery, Nanjing Hospital Affiliated to Nanjing University of Chinese Medicine, Nanjing Second Hospital, Nanjing, 210003 Jiangsu Province China

**Keywords:** Gastric cancer, LINC01094, CDKN1A, RUNX1, RNA-binding protein

## Abstract

**Background:**

Accumulating studies have focused on long noncoding RNAs (lncRNAs) because of their regulatory effects on multiple cancers. However, the biological functions and molecular mechanisms of lncRNAs in gastric cancer (GC) remain to be elucidated in depth.

**Methods:**

Long intergenic nonprotein coding RNA 1094 (LINC01094), a differentially expressed lncRNA between GC tissues and adjacent normal tissues, was identified. Moreover, gain- and loss-of-function experiments in vitro and in vivo were carried out. To understand the mechanisms underlying the regulatory effects of LINC01094, we performed RNA pull-down assays, RNA immunoprecipitation assays, chromatin immunoprecipitation assays, luciferase reporter assays, etc.

**Results:**

LINC01094 was markedly upregulated in GC tissues and cell lines, and LINC01094 upregulation was positively correlated with GC malignant behaviours in vitro and in vivo. Mechanistically, LINC01094 downregulated the expression of CDKN1A by interacting with RNA binding motif single stranded interacting protein 2 (RBMS2) and histone deacetylase 1 (HDAC1). Additionally, LINC01094 was confirmed to sponge miR-128-3p and participate in the LINC01094-miR-128-3p-RUNX family transcription factor 1 (RUNX1) feedback loop. Finally, Ro 5-3335, a validated RUNX1 inhibitor, was explored for anticancer drug development in GC.

**Conclusions:**

The LINC01094-miR-128-3p-RUNX1 feedback loop downregulates CDKN1A and promotes GC cooperatively with RBMS2 and HDAC1. Furthermore, Ro 5-3335 may hold promising therapeutic potential in the treatment of GC. Hence, our study found an oncogenic lncRNA, LINC01094, which could be a promising target for cancer treatment and diagnosis.

**Graphical Abstract:**

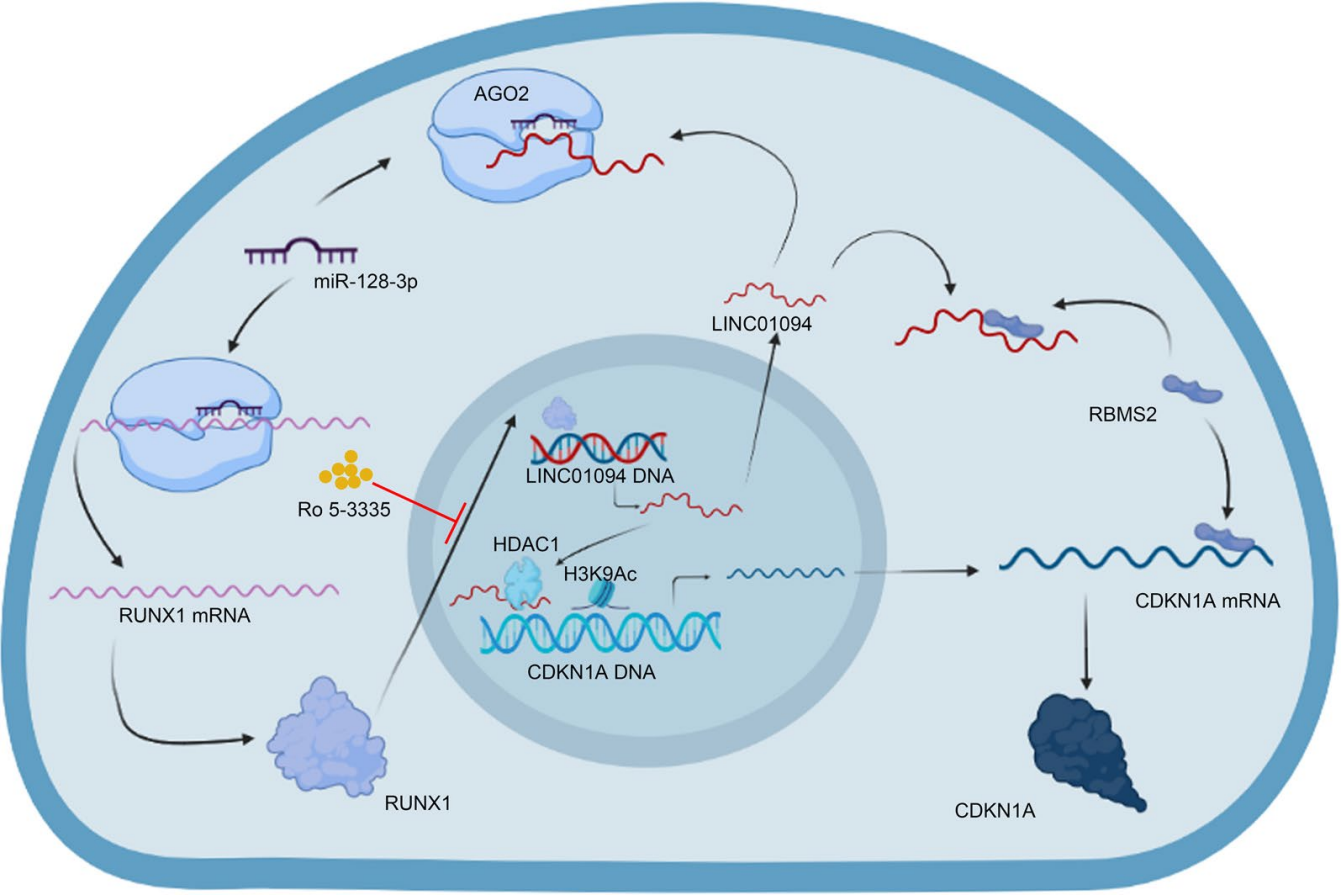

**Supplementary Information:**

The online version contains supplementary material available at 10.1186/s13062-024-00582-y.

## Introduction

As one of the most lethal malignant gastrointestinal tumors, gastric cancer (GC) accounted for at least one million new cases and approximately 760,000 deaths worldwide in 2020 [1]. Despite developments and improvements in GC diagnosis and treatment resulting from many related studies, surgical resection remains the only potentially curative option for GC patients [2]. Thus, it is essential to seek pivotal molecular targets and potential regulatory networks of GC.

Long noncoding RNAs (lncRNAs) are RNA molecules that exceed 200 nucleotides (nt) in length and are known to possess limited protein-coding potential [3]. Accumulating evidence indicates that lncRNAs can interact with multiple biological molecules and play roles as parts of complex regulatory networks in various diseases, including cancers [4–6]. Although the abilities of lncRNAs to function as competitive endogenous RNAs (ceRNAs) and sponge microRNAs have been extensively studied [7, 8], it is worth further exploring whether lncRNAs can bind proteins and play roles in diverse key biological processes. Some lncRNAs can recruit transcription factors and regulate chromosome status [9, 10], whereas some lncRNAs can affect posttranslational and posttranscriptional modifications [11, 12].

LINC01094 is a cancer-promoting molecule correlated with many classical signaling pathways, such as the Wnt/β-catenin pathway and PI3K/AKT pathway [13–15]. Many bioinformatic studies have predicted the potential of LINC01094 to affect angiogenesis, autophagy, epithelial-to-mesenchymal transition (EMT), and macrophage infiltration in GC [16–18]. Our study was designed to comprehensively assess the expression level and biological function of LINC01094. Then, we explored the underlying mechanisms based on its unique subcellular localization pattern in GC cell lines. Furthermore, we predicted the regulators of LINC01094 expression and conducted preliminary validation of a drug targeting this potential molecule.

## Methods

### Tissue samples

Eighty GC tissue samples and the corresponding adjacent normal tissues were obtained from the First Affiliated Hospital of Nanjing Medical University between April 2015 and April 2021. All patients were definitively diagnosed by at least two pathologists to have no history of preoperative chemoradiotherapy or other malignancies. The clinicopathological characteristics of the patients were obtained from medical records, and the survival status was ascertained through annual telephone interviews. After informed consent was obtained from each participant, our study was evaluated and approved by the Ethics Committee of the First Affiliated Hospital of Nanjing Medical University.

### Cell culture

The cell lines used in this study, including HEK-293 T, the normal human glandular epithelial cell line GES-1, and several common human GC cell lines, were provided by the Cell Center of Shanghai Institutes for Biological Sciences. HEK-293 T cells were cultured in Dulbecco’s modified Eagle’s medium (DMEM H-21 4.5 g/litre glucose) (Wisent, Canada), and AGS cells were cultured in Nutrient Mixture F-12 K medium (Wisent, Canada). The other aforementioned cell lines were cultured in RPMI 1640 medium. Foetal bovine serum (FBS; 10%, Wisent, Canada) was added to the aforementioned media along with 1% penicillin–streptomycin. All cell lines were incubated in a humidified atmosphere of 5% CO_2_ at 37 °C.

### Plasmid construction, siRNA transfection, and lentiviral infection

The human LINC01094 overexpression vector was constructed by inserting the corresponding cDNA sequence into the pcDNA3.1 vector (Obio, China), with the empty vector used as a control. Small interfering RNAs (siRNAs) against LINC01094 (GenePharma, China) were synthesized, with a scrambled siRNA used as a negative control. Lipofectamine 3000 (Invitrogen, USA) was used for transfection. The sequences of the LINC01094 cDNA and the siRNAs targeting LINC01094 and cyclin-dependent kinase inhibitor 1A (CDKN1A) were inserted into a lentiviral vector (GenePharma, China). Specific plasmids expressing and siRNAs targeting RNA binding motif single stranded interacting protein 2 (RBMS2), histone deacetylase 1 (HDAC1), CDKN1A, and RUNX family transcription factor 1 (RUNX1) were constructed as described above. Expression at different levels was evaluated by quantitative real-time reverse transcription polymerase chain reaction (qRT‒PCR) to validate the gene knockdown and overexpression efficiencies in the supplementary material (Fig. S1A, B and Fig. S3A–D). The related sequences were used in previous studies and are listed in the supplementary material [19–25].

### RNA extraction and qRT-PCR

Total RNA was extracted from cells and tissues by TRIzol reagent (Invitrogen, USA). The New Poly (A) Tailing Kit (Thermo Fisher Scientific, USA) and the Prime Script RT Reagent Kit (Takara, Japan) were used for the reverse transcription of miRNA and lncRNA/mRNA, respectively. Amplification was performed with SYBR Green Master Mix (Roche, China). LncRNA and mRNA expression were normalized to GAPDH expression. Related procedures were performed three times, and the 2^−ΔΔCT^ method was applied to evaluate expression levels. The primers used in this study are described in the supplementary material.

### Nuclear-cytoplasmic fractionation

The PARIS™ kit (Thermo Fisher Scientific, USA) was used for isolation of the nuclear and cytoplasmic fractions according to the manufacturer’s steps. After lysing and centrifuging the cells, we obtained the supernatant as the cytoplasmic fraction. Subsequently, we washed the pelleted nuclei with a Cell Fraction Buffer and collected the nuclear fraction.

### mRNA stability and protein stability analyzes

For mRNA stability analysis, we treated MKN45 cells with 5 μg/mL actinomycin D (ActD; Sigma-Aldrich, USA) and measured target gene expression at 2-h intervals (to 12 h). Cycloheximide (CHX; 50 μg/mL; Sigma‐Aldrich, USA) was used for protein stability analysis. The remaining procedures were similar to those described above.

### *Fluorescence *in situ* hybridization (FISH) assay*

Fluorescently labelled LINC01094 and miR-128-3p probes were constructed, and 4′,6-diamidino-2-phenylindole (DAPI) was applied to stain nuclei. The probe signals in GC cells and tissues were detected with a FISH kit (RiboBio, China). Images were obtained with confocal microscopy (Carl Zeiss, Germany).

### RNA pull-down assay

According to the instructions, we incubated a biotin-labeled LINC01094 probe (RiboBio, China) with streptavidin magnetic beads. After probe-coated beads were generated, we obtained MKN45 cell lysates, incubated them with the aforementioned beads overnight, and then washed the beads. Then, we performed silver staining with a silver staining kit (Thermo Fisher Scientific, USA). Furthermore, we performed mass spectrometry and western blotting to analyze the precipitated proteins.

### RNA immunoprecipitation (RIP) assay

Following the protocol of the Magna RIP RNA Binding Protein Immunoprecipitation Kit (Millipore, USA), we incubated magnetic beads with specific antibodies at 25 °C and mixed the GC cell lysates with the antibody-bead complexes at 4 °C. Then, qRT-PCR was applied to quantify the eluted bound RNAs.

### Chromatin immunoprecipitation (ChIP) assay

We purchased the Magna ChIP™ A/G Kit (Millipore, USA) for the ChIP assay. After fixing the cells with 1% formaldehyde and quenching the crosslinking reaction, we generated 200–1000-bp fragments of the crosslinked chromatin by sonication. The magnetic beads were incubated with related antibodies and cell lysates. Then, qRT-PCR was applied to analyze the eluted immunoprecipitated complexes.

### Triplex capture assay

We incubated MKN45 cell nuclei with 2 μg biotinylated triplex-forming oligonucleotides (TFOs) and subjected them to UV (365 nm) irradiation and sonication. After centrifugation of the nuclei, the supernatant was separated and incubated with streptavidin-magnetic beads (Thermo Fisher Scientific, USA) at 4 °C. Following DNA extraction and purification and qRT-PCR analysis, we washed the beads 5 times and then resuspended the eluted complexes. The primers used are described in the supplementary material.

### Luciferase reporter assay

The corresponding fragments were synthesized and subcloned into the luciferase reporter plasmid (Realgene, China). Various gene overexpression plasmids and siRNAs were cotransfected with the luciferase reporter plasmid. Then, we collected the cells and applied the Dual-Luciferase Assay Kit (Promega, USA). Firefly and Renilla luciferase activities were measured and are presented as relative luciferase activity values (firefly luciferase/Renilla luciferase).

### Western blot analysis

The western blot protocol was consistent with that used in our previous study, and the antibodies used are described in the supplementary material [26].

### Immunohistochemical (IHC) and hematoxylin and eosin (HE) staining

For IHC staining, paraffin-embedded tissues were dewaxed, rehydrated, and subjected to antigen retrieval. Specific antibodies were incubated with the sections, and expression levels were evaluated with diaminobenzidine (DAB) the next day.

Paraffin-embedded mouse lung tissues were sliced into 5-µm sections and stained with HE for pathologic analysis.

### Functional assays

#### Cell proliferation, migration, and invasion assays

To observe the effects on cell proliferation, migration, and invasion, Cell Counting Kit-8 (CCK-8), colony formation, wound healing, and transwell assays were performed as described in our previous study [26].

#### Flow cytometric analysis

The Annexin V-FITC/PI Apoptosis Detection Kit (Multisciences, China) and the Cell Cycle Analysis Kit (Multisciences, China) were applied to evaluate the apoptosis rate and the cell cycle, respectively, according to the corresponding protocols. The apoptosis rate was calculated as the sum of early apoptotic and late apoptotic cells. Cell cycle data were divided into G1, S as well as G2/M phases and analyzed by FlowJo (v10.8.1).

#### Animal experiments

Cell suspensions (0.1 ml, 1 × 10^6^ stable cells) were subcutaneously injected into four-week-old male nude mice (BALB/c) to establish the xenograft model. We measured the tumor volume every 2 days and weighed the xenografts after sacrificing the mice 2 weeks after cell injection. For the lung metastasis model, we injected cells into the caudal vein of nude mice, and metastatic tumors were detected by HE staining. The care of animals involved in this study was in accordance with the National Research Council's Guide for the Care and Use of Laboratory Animals.

#### GC organoid model

Human GC organoids model were constructed as described previously [27]. We transfected the LINC01094 shRNA and its negative control into the organoids to facilitate our investigation of the role of LINC01094 in GC progression. The growth of GC organoid was observed daily by microscope.

#### Bioinformatic and statistical analyzes

All DNA, RNA, and protein sequences used in this study can be searched in the National Center for Biotechnology Information (NCBI) database. Other public databases and the corresponding websites are listed in the supplementary material. SPSS (version 26.0) and GraphPad Prism (version 8.01) were used for statistical analysis. Student's t test, the Wilcoxon test, and the χ^2^ test were applied to analyze parametric variables, and the Kaplan‒Meier method was employed to analyze overall survival (OS). Quantitative data are presented as the means ± SDs. Each experiment was repeated independently in triplicate, and *p* < 0.05 was considered to indicate statistical significance.

## Results

### Identification and characteristics of LINC01094 in GC

**Fig. 1:**
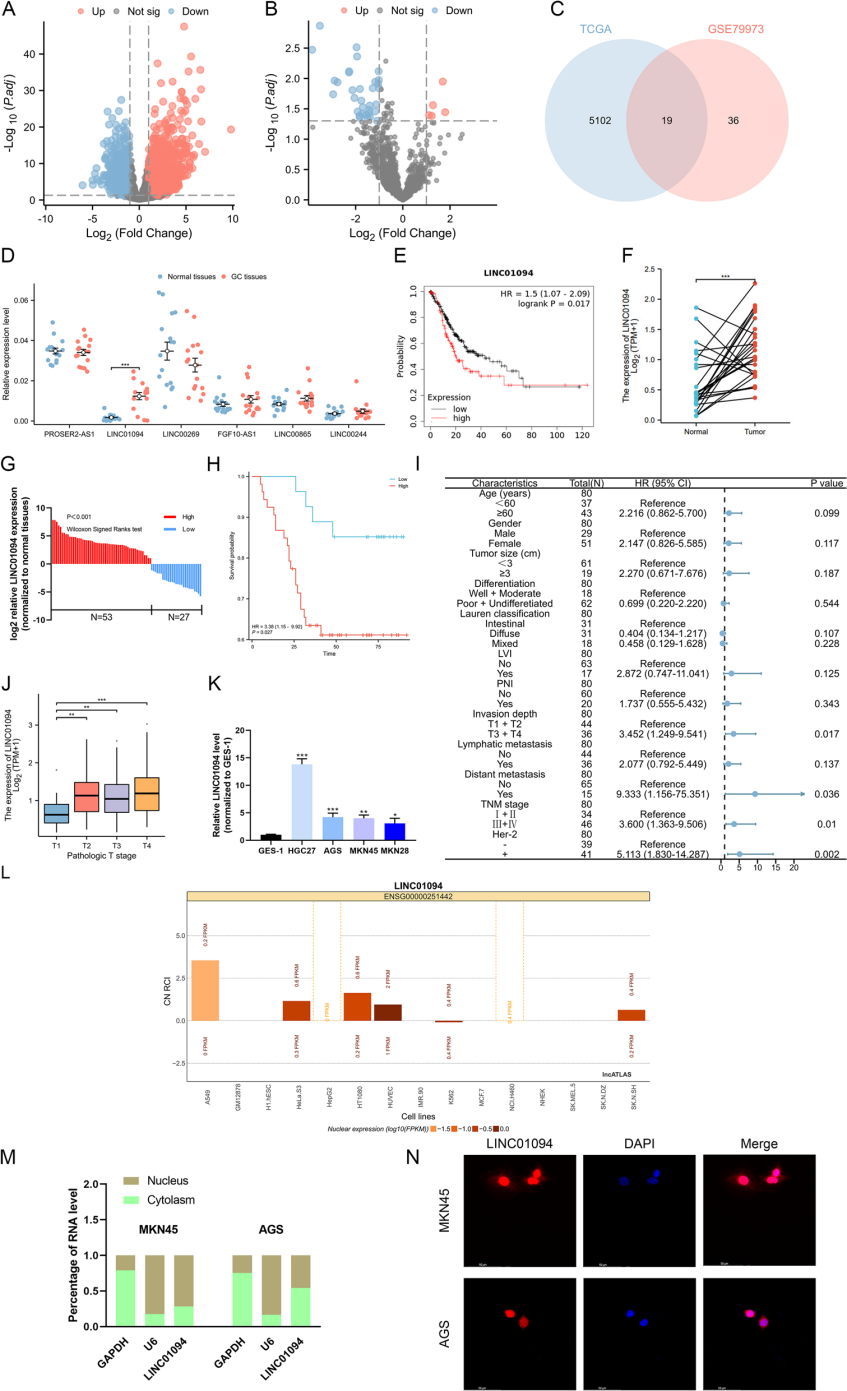
LINC01094 is upregulated in GC and correlated with poor prognosis. *Notes* **A** Differentially expressed lncRNAs in GC were shown by volcano plots according to the TCGA database. **B** Differentially expressed lncRNAs in GC were shown by volcano plots according to the GSE79973 dataset. **C** Nineteen lncRNAs upregulated in GC were identified after taking the intersection of both two databases.** D** LINC01094 was found significantly upregulated in GC tissues.** E** The association of LINC01094 with GC prognosis was predicted by online tools. **F** The expression of LINC01094 in 32 independent paired patient samples according to the TCGA STAD database.** G** qRT-PCR was used to detect the expression of LINC01094 in 80 GC tissues and paired adjacent normal tissues. **H** Survival analysis showed that LINC01094 predicted poor prognosis in GC. **I** The cox regression analysis showed that high expression of LINC01094 was correlated with advanced TNM stage and positive HER2 expression.** J** The association of LINC01094 expression with pathologic T stage was analyzed according to the TCGA STAD database. **K** qRT-PCR analysis was performed to show the LINC01094 expression in GC cells and normal gastric epithelium cell line (GES-1). **L, M** Subcellular localization of LINC01094 was presented by lncATLAS **(L)**, qRT-PCR **(M)**. **N** The FISH assay showed the localization of LINC01094 in GC cells

Analysis of the TCGA STAD dataset and a GEO dataset (GSE79973) revealed 19 lncRNAs upregulated in GC (Fig. 1A–C). Among the six survival-associated lncRNAs in GC predicted by the Kaplan‒Meier Plotter website, LINC01094 was identified as overexpressed in GC after qRT‒PCR validation (n = 16) (Fig. 1D, E). This pattern was consistent with that in paired tumor and adjacent tissues (n = 32) in the TCGA STAD dataset (Fig. 1F). To explore the relationships between the LINC01094 expression level and clinicopathological characteristics, qRT‒PCR was performed in a larger sample (n = 80) (Fig. 1G). Statistical analysis showed that a high LINC01094 expression level was positively correlated with invasion depth, distant metastasis, advanced TNM stage, and poor prognosis (Fig. 1H, I). Moreover, higher LINC01094 expression may predict a higher T stage, based on analysis of the TCGA STAD dataset (Fig. 1J), prompting us to hypothesize the effects of LINC01094 on GC cell malignant behaviors. In subsequent functional assays, we measured the LINC01094 expression level in GC cell lines and found that LINC01094 expression was higher in GC cell lines (Fig. 1K). Based on these results, we chose two GC cell lines with intermediate LINC01094 expression levels (MKN45 and AGS) to explore the subcellular localization of LINC01094. In contrast to the prediction of lncATLAS (Fig. 1L), LINC01094 was localized in both the cytoplasm and nucleus of GC cells, as determined by nuclear-cytoplasmic isolation and FISH assays (Fig. 1M, N).

### *LINC01094 promotes malignant behaviors of GC cells *in vitro* and *in vivo

**Fig. 2:**
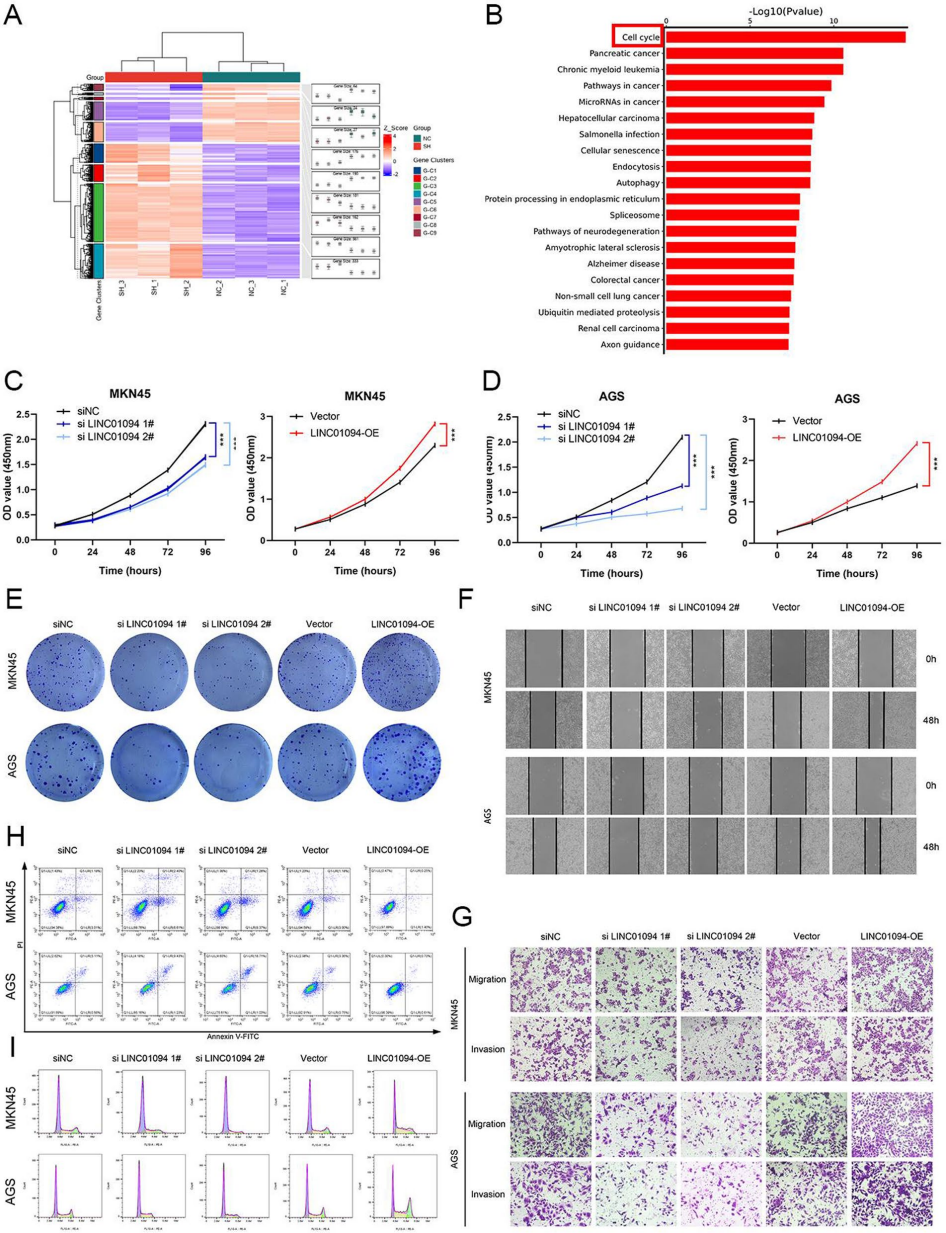
LINC01094 regulates GC cell cycle and modulates the malignant behaviors of GC cells in vitro. *Notes A* The heatmap showed the mRNAs differentially expressed in three pairs of group KD NC and group LINC01094 KD cells. **B** The molecular pathway enrichment analysis of differentially expressed genes was conducted to explore the mechanism underlying LINC01094 in GC development. **C, D** The growth curves of cells were evaluated by CCK-8 assays after knocking down **(C)** and overexpressing **(D)** LINC01094 in MKN45 and AGS cells.** E** Colony formation assays were conducted to evaluate cell proliferation.** F, G** Wound healing assays **(F)** and transwell assays (**G**) were conducted to evaluate cell migration and invasion. **H** Flow cytometric analysis presented apoptosis rates of cells.** I** Flow cytometric analysis was performed to analyze cell cycle progression. Knockdown and overexpression of LINC01094 inhibited and facilitated G1 to S transition, respectively

To evaluate the biological effects of LINC01094 on GC cells, we transfected LINC01094 shRNAs and its negative control into GC cells following by subsequent transcriptome sequencing (Fig. 2A). After molecular pathway enrichment analysis for differentially expressed genes, we noticed that there exists the most significant association between LINC01094 and the process of cell cycle (Fig. 2B). CCK-8 and colony formation assays were performed to evaluate GC cell proliferation, while wound healing and transwell assays were used to assess the migratory and invasive capacities of GC cells. Knockdown of LINC01094 suppressed and overexpression of LINC01094 promoted GC cell proliferation, migration, and invasion (Fig. 2C–G and Fig. S1C–E). Additionally, flow cytometric analysis indicated that higher LINC01094 expression was associated with a lower apoptosis rate and attenuated G1/S arrest (Fig. 2H, I and Fig. S1F, G).

**Fig. 3:**
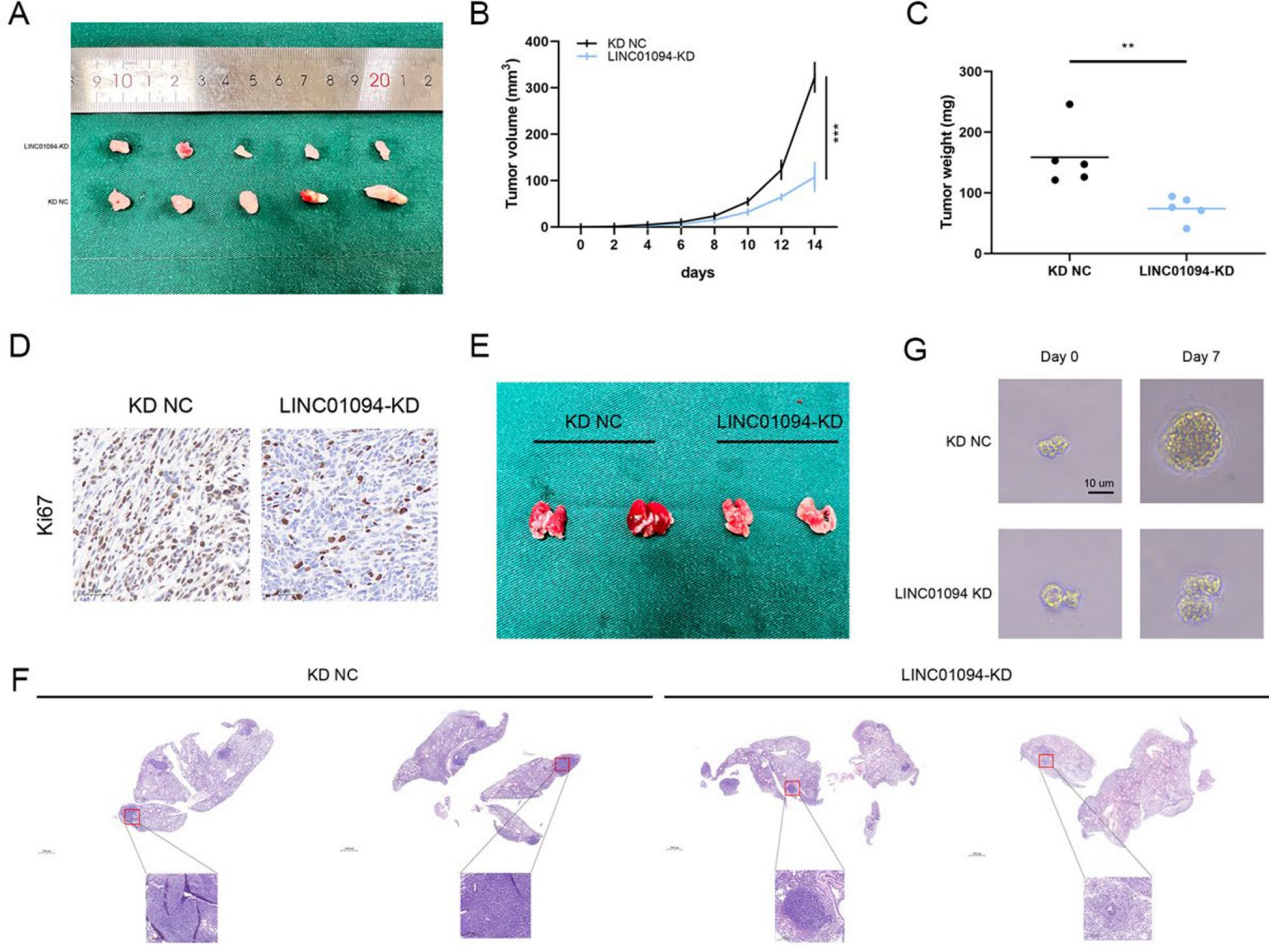
LINC01094 facilitates the tumorigenesis and metastasis of GC cells in vivo. *Notes* **A** Xenograft tumor models showing that LINC01094 knockdown inhibited GC growth in vivo. **B, C** The tumor size **(B)** and weight **(C)** in different groups were analyzed. **D** The expression of Ki67 from the xenografts was measured by IHC (scale bar: 50 μm).** E, F** Representative images of lung metastasis and HE staining of the specimen were shown. **G** We observed that LINC01094 knockdown significantly inhibited the growth of the organoid models

To assess the influence of LINC01094 on GC in vivo, MKN45 cells stably transduced with LV-si-LINC01094 (LINC01094-KD) or the corresponding negative control construct (KD NC) were subcutaneously injected into BALB/c nude mice. Significantly lower volume and weight, as well as a decreased Ki67 level, were observed in LINC01094-KD group tumors compared with KD NC group tumors (Fig. 3A–D and Fig. S1H). In the lung metastasis model, fewer metastatic nodules were detected in the LINC01094-KD group, and this finding was validated by HE staining (Fig. 3E, F). At last, we constructed human GC organoid models to validate the biological functions of LINC01094. We observed that LINC01094 knockdown remarkbly suppressed the growth of the organoid models (Fig. 3G).

### LINC01094 reduces CDKN1A mRNA stability by inhibiting the binding of RBMS2 to the CDKN1A 3′-untranslated region (UTR)

**Fig. 4:**
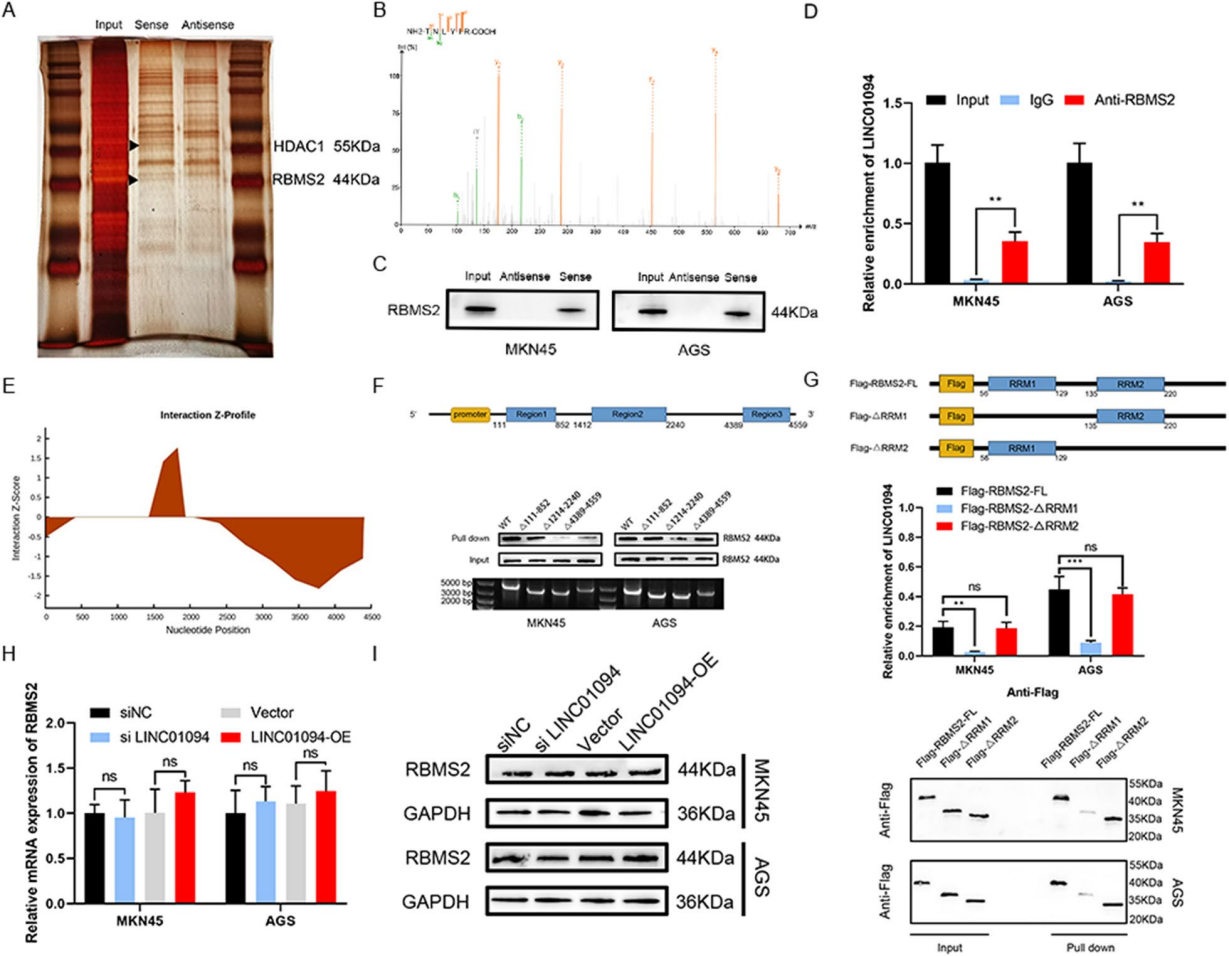
LINC01094 binds directly with RBMS2. *Notes* **A** Silver staining of LINC01094 pull-down was shown. **B, C** The secondary mass spectrometry of RBMS2 **(B)** and western blots **(C)** were presented to verify that RBMS2 was enriched in the LINC01094 probe. **D** RIP assay showed that RBMS2 was precipitated with LINC01094 in GC cell lysates. **E** The binding regions of LINC01094 with RBMS2 were predicted by the catRAPID database. **F** The schematic diagram of the truncated fragment of LINC01094 overexpression plasmids was presented (top). After GC cells were transfected with wild-type or truncated LINC01094 overexpression plasmids, RNA pull-down was performed with LINC01094-specific probes (bottom). **G** The schematic diagram of different flag-labeled recombinant RBMS2 was shown (top). The full-length or truncated forms of flag-labeled recombinant RBMS2 were incubated with cell lysates and the LINC01094 levels were detected by qRT-PCR (middle). RNA pull-down experiments made use of LINC01094-specific probes against full-length or truncated forms of flag-tagged recombinant RBMS2 proteins (bottom). **H, I** Knockdown or overexpression of LINC01094 had no effect on the expression of RBMS2 at both mRNA **(H)** and protein levels **(I)**

**Fig. 5:**
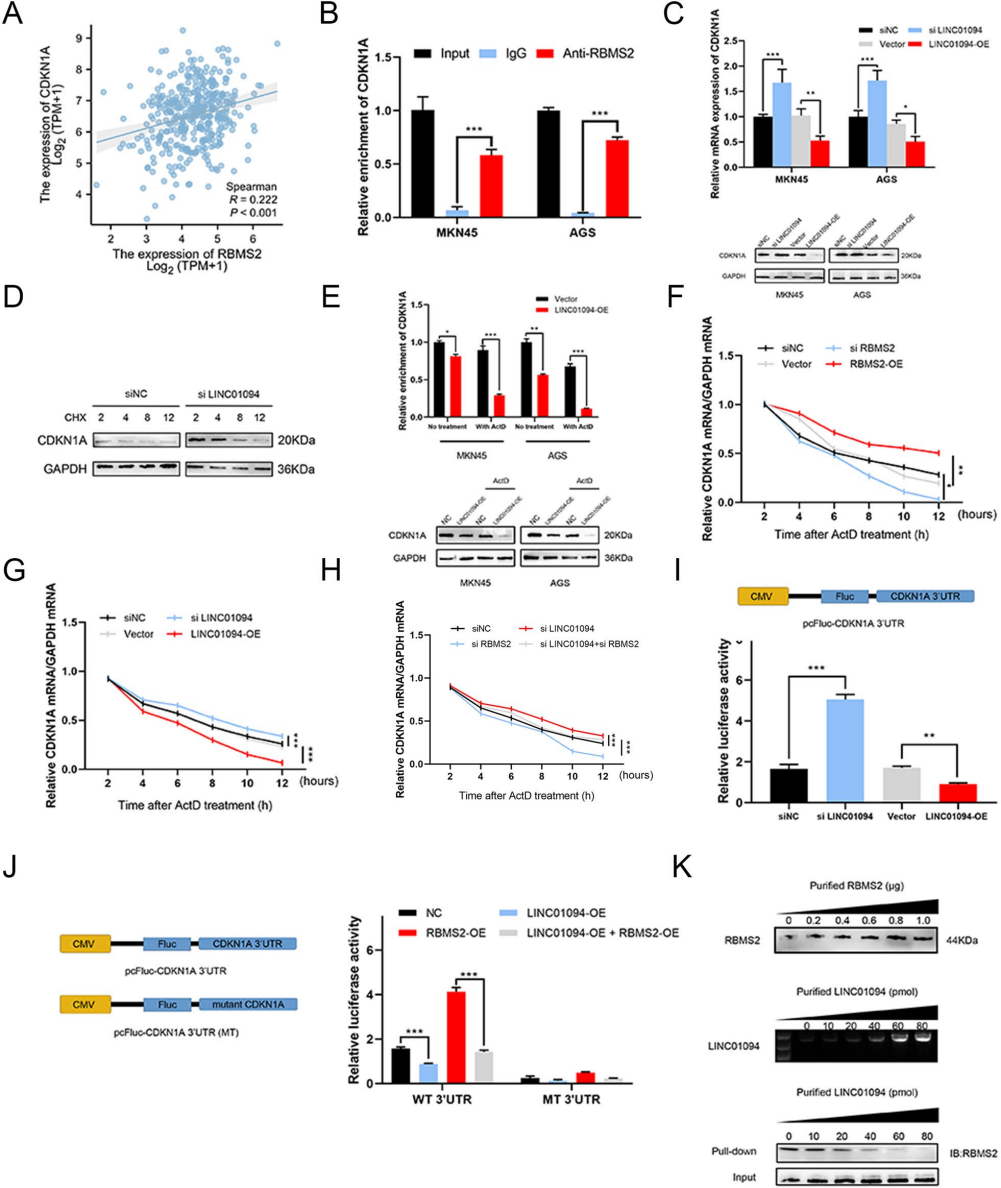
LINC01094 decreases CDKN1A mRNA stability by competitively interacting with RBMS2. *Notes* **A** The expression of RBMS2 was positively correlated with that of CDKN1A in the TCGA STAD database. **B** RIP assay showed that RBMS2 was precipitated with CDKN1A mRNA in GC cell lysates. **C** qRT-PCR and western blot showed that the expression of RBMS2 was positively correlated with that of CDKN1A in GC cells. **D** LINC01094 knockdown MKN45 cells were treated with the translation inhibitor, CHX, after which lysates were prepared at the indicated times for western blot. **E** Overexpression of LINC01094 reduced the half-life of CDKN1A mRNA. **F–H** Cells with different treatments were incubated with ActD for the indicated times, followed by qRT-PCR. RBMS2 could increase CDKN1A mRNA stability **(F)** and LINC01094 had adverse effects **(G)**. RBMS2 could rescue the effects of LINC01094 on CDKN1A mRNA stability **(H)**. **I** Knockdown of LINC01094 showed increased p21 mRNA stability and overexpression of LINC01094 showed adverse results, as indicated by altered CDKN1A 3′UTR reporter activity. **J** Luciferase reporters containing the CDKN1A 3′UTR region and ARE mutant region were constructed (left). Relative luciferase activity was measured (right). **K** Efficiencies of purified RBMS2 protein and purified LINC01094 were verified. Different amounts of purified LINC01094 competed with the RBMS2-CDKN1A complex in a dose-dependent manner

**Fig. 6:**
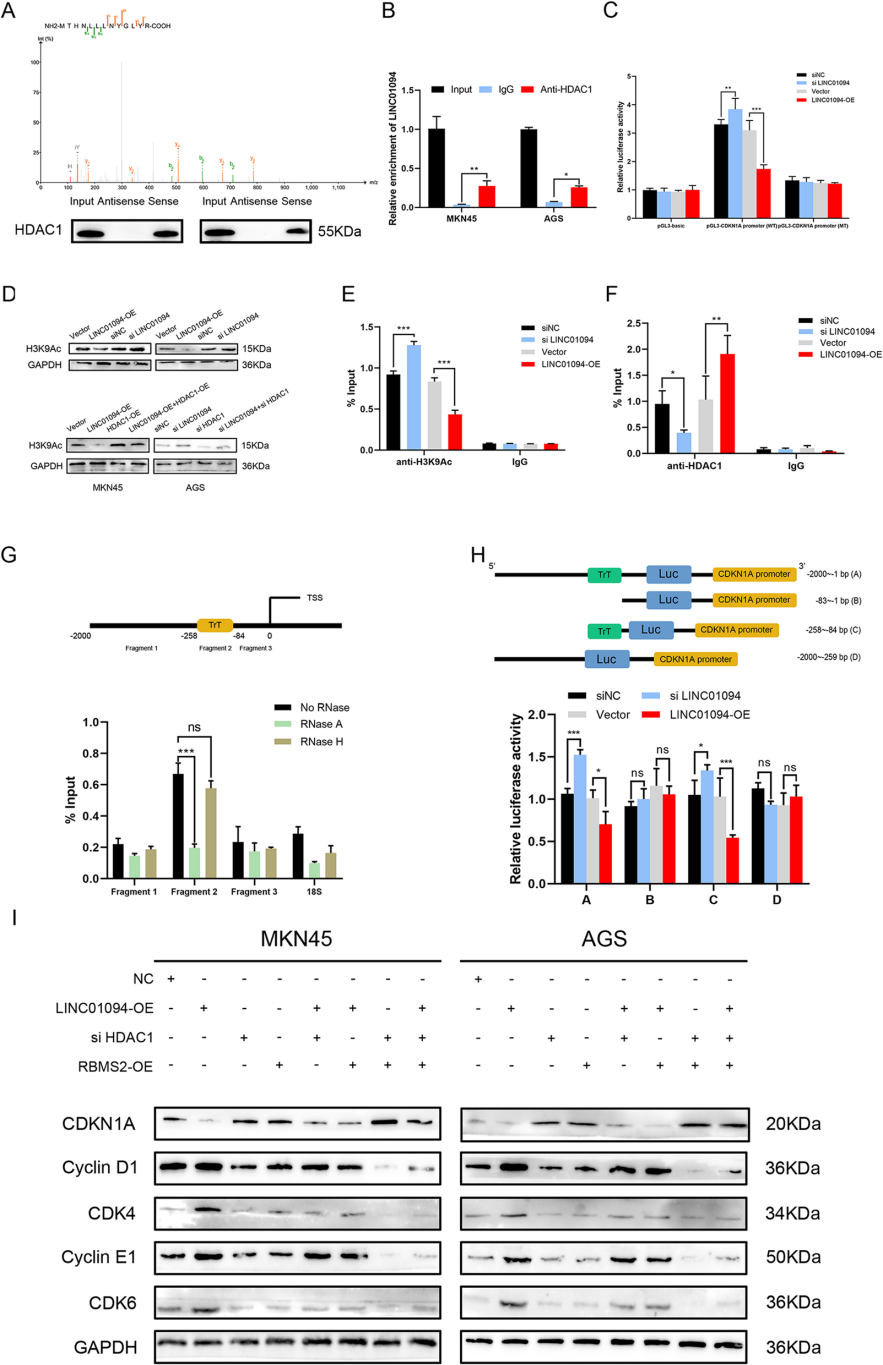
LINC01094 inhibits CDKN1A mRNA transcription by interacting with HDAC1. *Notes A* The secondary mass spectrometry of HDAC1 and western blots were presented to verify that HDAC1 was enriched in the LINC01094 probe. **B** RIP assay showed that HDAC1 was precipitated with LINC01094 in GC cell lysates. **C** Dual-luciferase reporter assays showed that LINC01094 inhibited the transcriptional activity of the CDKN1A promoter. **D** LINC01094 inhibited H3K9Ac expression (top), which could be rescued by HDAC1 (bottom). **E, F** ChIP-qPCR showed that LINC01094 silencing increased the occupancy of H3K9Ac **(E)** and HDAC1 **(F)** on the CDKN1A promoter in HEK-293 T cells. **G** Chromatin was pretreated with RNase A or RNase H and triplex-qPCR suggested that LINC01094 formed a triplex structure within the CDKN1A promoter in HEK-293 T cells. **H** We evaluated wild-type and different truncated CDKN1A promoters’ luciferase activities by luciferase assay in HEK-293 T cells with different treatments. **I** The expression of CDKN1A and its downstream molecules was evaluated by western blotting in GC cells following the treatments indicated

For further analysis, RNA-binding proteins were speculated to be associated with the effects of LINC01094, and an RNA pull-down assay was performed with MKN45 cells to identify the LINC01094-interacting proteins. As the silver staining assay showed, two bands at 44 kDa and 55 kDa exhibiting significant enrichment were observed (Fig. 4A) and were identified to correspond to RBMS2 and HDAC1 by mass spectrometry and western blotting (Figs. 4B, C, 6A). Then, we performed the RIP assay and obtained the same result (Figs. 4D, 5B). Regarding the subcellular localization of LINC01094, we assumed that LINC01094 interacted with RBMS2 and HDAC1 in the cytoplasm and nucleus, respectively. The three potential regions where LINC01094 may bind to RBMS2 (111–852 nt, 1412–2240 nt, and 4389–4559 nt) were predicted by the catRAPID database (Fig. 4E). Following RNA pull-down assays and western blotting, plasmids containing individual truncations of these regions were constructed and transfected into MKN45 and AGS cells. It was confirmed that LINC01094 primarily interacts with RBMS2 via its region spanning nucleotides 1412–2240 nt (Fig. 4F). To identify the specific structural domain of RBMS2 responsible for its binding to LINC01094, RBMS2 mutants with truncated individual structural domains were constructed. The subsequent RIP and RNA pull-down assays revealed that RNA recognition motif 1 (RRM1) of RBMS2 contributed greatly to the interaction of RBMS2 with LINC01094 (Fig. 4G). RBMS2 was reported to directly bind to AU-rich elements (AREs) in the 3′-UTR to improve the stability of CDKN1A mRNA [10, 21, 25]. The production of CDKN1A, namely p21, is considered as an important part of the p53 pathway and can be involved in tumor development by regulating processes such as the cell cycle, which may be the downstream molecule of LINC01094 [28, 29].

For neither the mRNA nor the protein expression of RBMS2 was affected (Fig. 4H, I), we speculated that LINC01094 plays a role by competing with CDKN1A for binding to RBMS2. After preliminary molecular correlation analysis between RBMS2 and CDKN1A in the TCGA STAD dataset (Fig. 5A), RIP assays, qRT‒PCR, and western blotting were performed and showed that RBMS2 can also bind to CDKN1A and increase CDKN1A expression in GC (Fig. 5B, C). Then, differently treated cells were treated with an inhibitor of translation (CHX) or a transcription (ActD). LINC01094 may modulate CDKN1A expression by affecting CDKN1A mRNA stability instead of CDKN1A protein stability, consistent with the effect of RBMS2 (Fig. 5D, E). Further analysis showed that the CDKN1A mRNA half-life was positively correlated with RBMS2 expression (Fig. 5F) and negatively correlated with LINC01094 expression (Fig. 5G). RBMS2 knockdown rescued the effect of LINC01094 downregulation on decreasing the CDKN1A mRNA half-life (Fig. 5H). We constructed reporter vectors containing the p21 3′-UTR and p21 3′-UTR with mutant AREs for luciferase reporter assays and obtained similar results (Fig. 5I, J). For the competition assay, biotin-labeled CDKN1A mRNA was incubated with purified RBMS2 protein containing different amounts of purified LINC01094. LINC01094 was found to promote CDKN1A decay in a dose-dependent manner by competitively binding to RBMS2 (Fig. 5K).

Despite the numerous studies in other tumors [10, 21, 25], the function of RBMS2 in GC has not been reported. Significant differences in RBMS2 expression between tumor tissues and adjacent normal tissues were found by qRT-PCR and western blotting (Fig. S2A, B). In contrast to the findings regarding LINC01094, RBMS2 was found to have obvious tumor-suppressive effects in subsequent functional experiments in vitro (Fig. S2C–I and Fig. S3E–J).

### LINC01094 decreases CDKN1A transcription by recruiting HDAC1 to the CDKN1A promoter

HDAC1, a histone deacetylase, is correlated with the dysregulation of tumor suppressor genes in several malignant tumors [30, 31], and we identified it as a protein that binds to LINC01094 (see above). Considering previous studies on RNA‒DNA triplexes [32–34] and the correlations between HDAC1 and CDKN1A [35, 36], we hypothesized that LINC01094 may play a key bridging role in recruiting HDAC1 to the CDKN1A promoter. To validate this hypothesis, a promoter luciferase assay was performed and indicated that LINC01094 could suppress the transcriptional activity of the CDKN1A promoter (Fig. 6C). A report indicated that H3 lysine-9 (H3K9) is the key site in the interaction between HDAC1 and CDKN1A in epidermal progenitor cells [37]. We examined the expression level of H3K9Ac after different treatments (Fig. 6D). Further ChIP assays showed a positive correlation between the LINC01094 expression level and the HDAC1 enrichment level. An inverse correlation was found between LINC01094 expression and H3K9 acetylation (H3K9Ac), the substrate of HDAC1 (Fig. 6E, F). To gain deeper insights into RNA–DNA triplex formation, we utilized the LongTarget website to predict potential binding sites between LINC01094 and the CDKN1A promoter. The triple target site (TrTs) within the CDKN1A promoter was visualized and confirmed through a triplex capture assay (Fig. 6G). Additionally, the luciferase reporter containing the mutant CDKN1A promoter could not respond to changes in LINC01094 expression when the TrTs was not present (Fig. 6H).

### LINC01094 exerts biological effects by downregulating CDKN1A

**Fig. 7:**
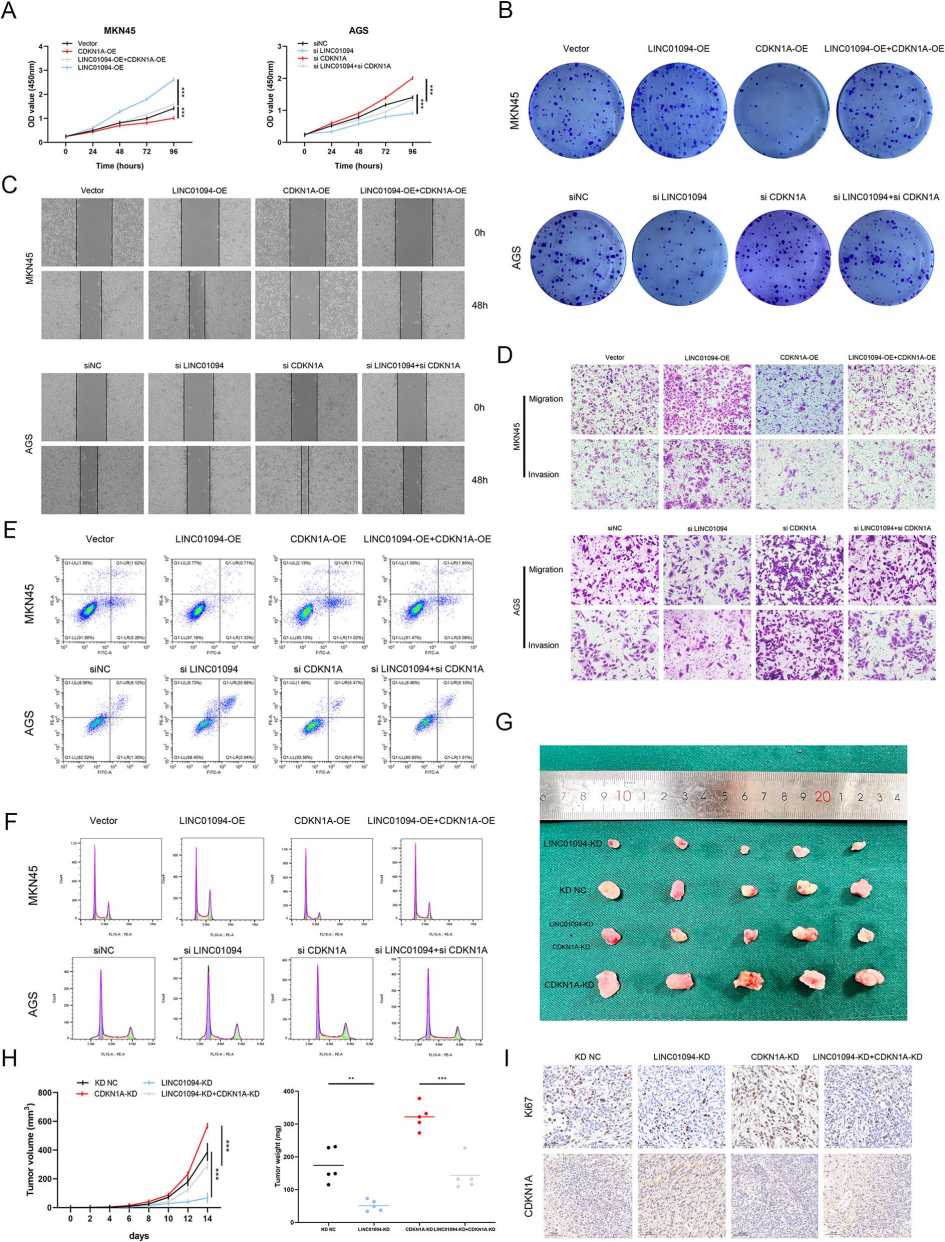
LINC01094 promotes malignant behaviors in GC by downregulating CDKN1A. *Notes A–I* CDKN1A overexpression reverses the effects of LINC01094 overexpression on regulating cell proliferation **(A, B)**, migration, invasion **(C, D)**, apoptosis **(E)**, and cell cycle **(F)** in vitro as well as tumor growth in vivo **(G–I)**

The above results demonstrated that LINC01094 could regulate CDKN1A expression in an intricate manner. The key downstream molecule of LINC01094, CDKN1A, known as p21, is an important component of the p53 signaling pathway and has potent regulatory effects on the cell cycle, apoptosis, and other processes [38, 39]. Similarly, a comparable effect has been observed in GC. We further validated the role of CDKN1A through qRT-PCR and survival analysis. The findings revealed that CDKN1A is significantly downregulated in GC tissues, and its expression demonstrates a positive correlation with patient prognosis (Fig. S4). Western blotting revealed that LINC01094 knockdown or overexpression modulated the expression of CDKN1A and its downstream molecules and that RBMS2 or HDAC1 expression may affect the aforementioned effect of LINC01094 (Fig. 6I). Then, we performed rescue experiments and observed that the attenuation of malignant behaviors after downregulating LINC01094 was partially reversed by CDKN1A knockdown, while the enhanced malignant phenotypes resulting from LINC01094 overexpression were attenuated by CDKN1A upregulation (Fig. 7A–I and Fig. S3L–S). In brief, this series of experiments indicated that the cancer-promoting effects of LINC01094 in GC depend greatly on the LINC01094-RBMS2/HDAC1-CDKN1A axis.

### LINC01094 upregulates RUNX1 by sponging miR-128-3p

**Fig. 8:**
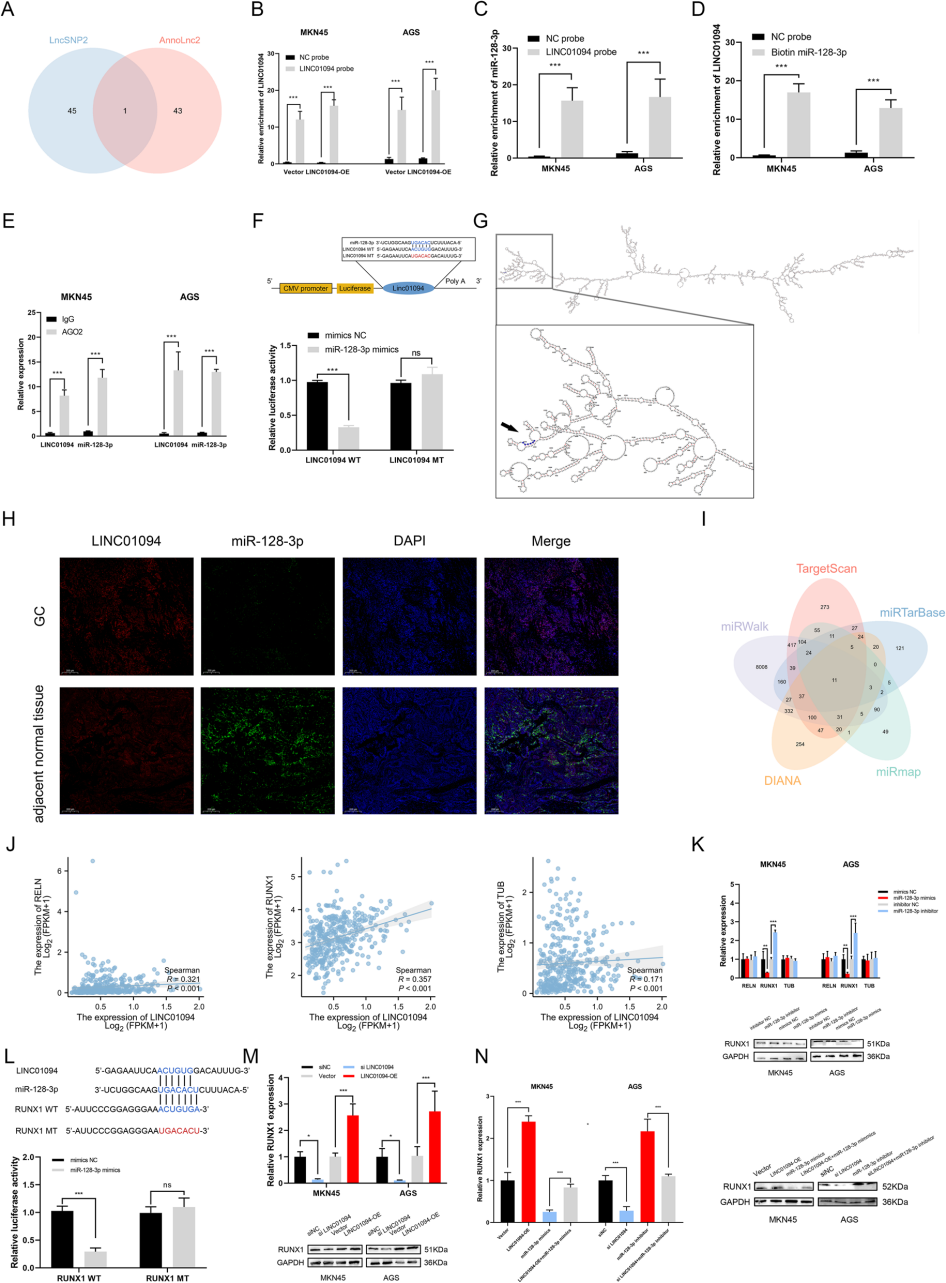
LINC01094 serves as a sponge for miR-128-3p and RUNX1 is a direct target of miR-128-3p. *Notes* **A** Venn diagram showed the overlap of the target miRNAs of LINC01094 predicted by LncSNP2 and AnnoLnc2. **B** The efficiency of the LINC01094 probe in GC cells was validated by qRT-PCR. **C** The relative levels of miR-128-3p in GC cell lysates were detected after the RNA pull-down assay. **D** After biotinylated miRNA pull-down in GC cells, qRT-PCR was performed and showed LINC01094 could bind with miR-128-3p. **E** Anti-Ago2 RIP was performed using GC cells followed by qRT-PCR to detect LINC01094 and miR-128-3p. **F** A schematic of the wild-type (WT) and mutant (MUT) LINC01094 luciferase reporter vectors was presented (top). A luciferase reporter assay was used to confirm the interaction between LINC01094 and miR-128-3p (bottom). **G** The combined area between LINC01094 and miR-128-3p was visualized according to AnnoLnc2 database. **H** FISH results showed the colocalization of LINC01094 and miR-128-3p in GC and adjacent normal tissues from patients. Scale bar = 200 μm. **I** Venn diagram showed 11 genes that are putative miR-128-3p targets predicted by five algorithms (Targetscan, miRTarBase, miRmap, miRWalk, and DIANA). **J** The association between the expression of RELN (left), RUNX1 (middle), TUB (right) and that of LINC01094 was shown according to the TCGA STAD database. **K** The expression of RUNX1 was correlated with that of miR-128-3p. **L** A schematic of the wild-type (WT) and mutant (MUT) RUNX1 luciferase reporter vectors was shown (top). A luciferase reporter assay in HEK-293 T cells was used to confirm the interaction between RUNX1 and miR-128-3p (bottom). **M** The expression of RUNX1 in GC cells upon LINC01094 overexpression or knockdown were examined. **N** The expression levels of RUNX1 were measured with co-transfecting LINC01094 siRNA and miR-128-3p inhibitor or LINC01094 overexpression vector and miR-128-3p mimics

It has been widely shown that lncRNAs can regulate downstream genes by functioning as miRNA sponges [40, 41]. We applied LncSNP2 and AnnoLnc2 to identify the target of LINC0194, and miR-128-3p was found in both of these online databases (Fig. 8A). After qRT‒PCR validation of the biotin-labelled LINC01094 probe efficiency, miR-128-3p was demonstrated to be significantly enriched in the sponged complexes in MKN45 and AGS cells (Fig. 8B, C). To exclude the possibility of false positives resulting from indirect binding, a biotin-labelled miR-128-3p probe was used in the RNA pull-down assay, and LINC01094 showed marked enrichment with this probe compared with the control probe (Fig. 8D). For miRNAs could bind to Argonaute-2 (AGO2) as RNA-induced silencing complex (RISC) components [42, 43], an RIP assay was performed and both LINC01094 and miR-128-3p were pulled down by the anti-AGO2 antibody (Fig. 8E). Additionally, the potential binding region was visualized, and a dual-luciferase reporter assay was conducted to further characterize the interaction. The miR-128-3p mimics attenuated the luciferase activity of wild-type LINC01094 but not that of mutant-type LINC01094 (Fig. 8F–G). Moreover, the FISH results showed that LINC01094 was primarily and highly expressed in the GC tissues, while miR-128-3p was mostly located in the adjacent normal tissue. The relationship of co-localization existed between LINC01094 and miR-128-3p (Fig. 8H).

We next sought to identify the target gene of LINC01094 and miR-128-3p. We intersected the predictions of multiple databases to predict the potential downstream gene (Fig. 8I). Through this predictive screening approach, three genes significantly associated with GC prognosis and LINC01094 expression were chosen as the most likely candidates (Fig. 8J). The qRT‒PCR and western blot results showed a negative correlation between miR-128-3p and RUNX1 expression (Fig. 8K). In the luciferase assay, the miR-128-3p mimics decreased luciferase activity in cells transfected with the WT 3′-UTR rather than the MT 3′-UTR of RUNX1 (Fig. 8L). Next, we investigated the association of LINC01094 with RUNX1 and examined whether the effect of LINC01094 on RUNX1 could be reversed by miR-128-3p (Fig. 8M, N).

### LINC01094 is upregulated by RUNX1

**Fig. 9:**
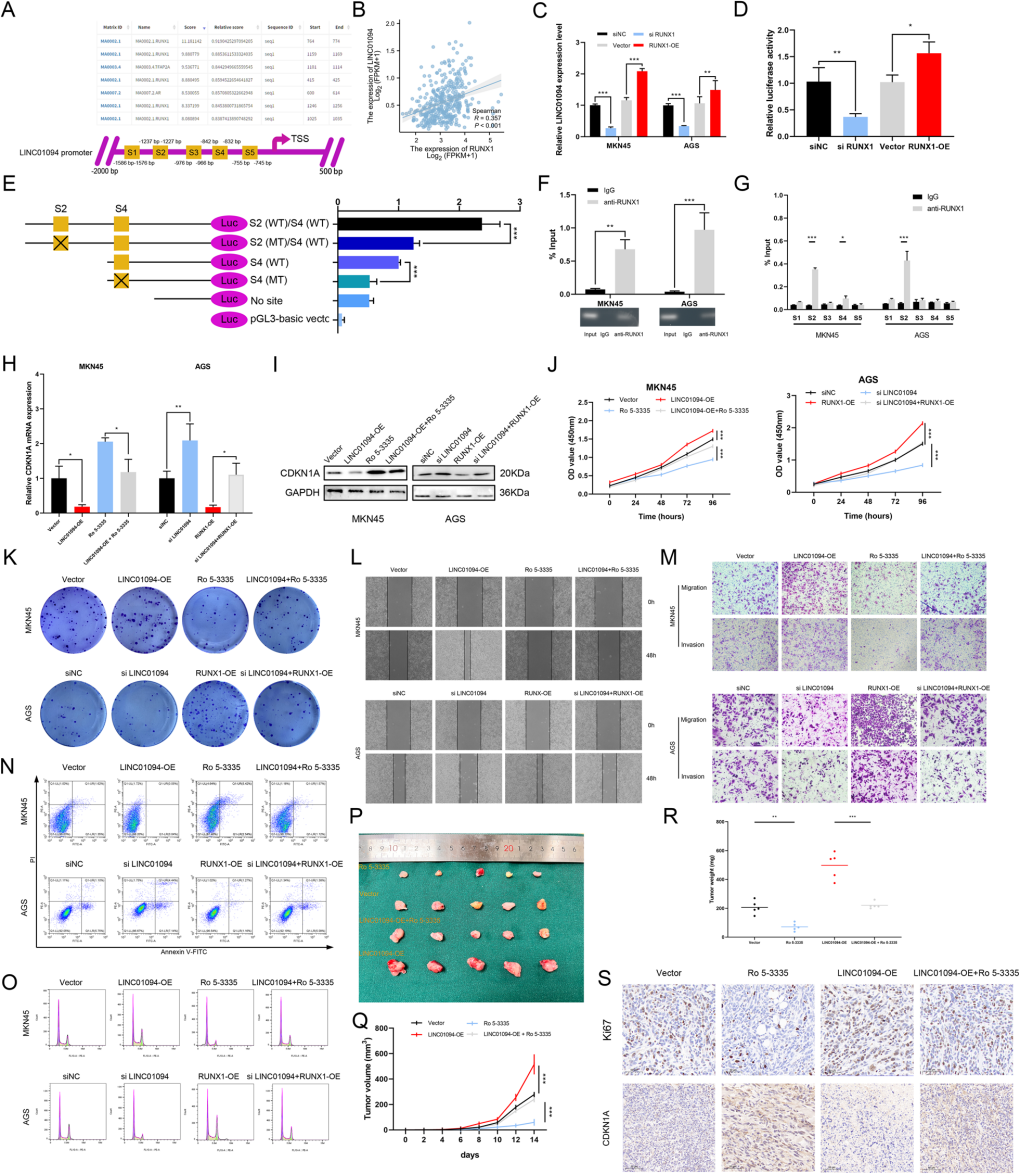
RUNX1 promotes LINC01094 expression and the regulative effect could be inhibited by Ro 5–3335. *Notes* **A** RUNX1 was predicted by the JASPAR database to bind with LINC01094 (top) and the potential binding region was visualized (bottom). **B** The expression of LINC01094 was positively correlated with that of RUNX1 according to the TCGA STAD database. **C** The association between the expression of LINC01094 and that of RUNX1 was validated by qRT-PCR. **D** A luciferase reporter assay was used to confirm that RUNX1 could promote the transcription of LINC01094. **E** The binding region between RUNX1 and LINC01094 was examined by a luciferase assay. **F, G** ChIP-qPCR indicated that RUNX1 could promote LINC0194 transcription by binding with site 2 and site 4 in the LINC01094 promoter. **H, I** RUNX1 could regulate CDKN1A expression by affecting LINC01094. **J–R** The effects of LINC01094 on malignant behaviors in vitro **(J–O)** and in vivo **(P–S)** could be affected by RUNX1 expression

Interestingly, RUNX1 was identified as the most likely transcription factor targeting LINC01094 by analysis of the JASPAR database (Fig. 9A). RUNX1 regulates oncogenes and has significant impacts on haematologic malignancies [44, 45]. In light of our investigation into the relationship between LINC01094 and RUNX1 (Fig. 9B), we promptly formulated a hypothesis regarding a feedback loop. We applied qRT‒PCR to preliminarily confirm this hypothesis (Fig. 9C). The results of subsequent ChIP and luciferase assays confirmed this hypothesis (Fig. 9D, F), indicating that RUNX1 may play a critical role in regulating CDKN1A. Based on the JASPAR database prediction, the five sites located in the LINC01094 promoter region had a relatively high probability of binding directly to RUNX1 (Fig. 9A). Different LINC01094 promoter fragments were amplified in the ChIP assay, with site 2 and site 4 exhibiting higher affinities for RUNX1 than the other sites. Subsequently, a luciferase assay was performed to further determine the binding site. Consistent with previous observations, site 2 and site 4 were required simultaneously during binding (Fig. 9E, G). Moreover, the effect of LINC01094 on CDKN1A expression was influenced by altering RUNX1 expression (Fig. 9H, I). A series of rescue experiments resulted in similar conclusions regarding the function of LINC01094 (Fig. 9J–S and Fig. S5).

## Discussion

As advancements in technology and understanding have progressed, various mechanisms of noncoding RNAs have recently been experimentally validated [46]. Multiple studies have indicated that individual noncoding RNAs can regulate tumor generation and progression in complex and sophisticated manners rather than via a single mechanism [47, 48].

To identify the target gene, we initially conducted differential expression and prognostic analyses. Following the validation of LINC01094's function in GC, we undertook an in-depth exploration of the underlying mechanisms. Then, the key molecules and the downstream pathway were identified. Finally, the inhibitor of RUNX1, Ro 5–3335, was used to disrupt the miR-128-3p-RUNX1-LINC01094-RBMS2/HDAC1-CDKN1A axis.

Competitive binding, a key action mechanism of LINC01094, occurs not only between noncoding RNAs and miRNAs but also between noncoding RNAs and proteins. Previous studies have indicated that competitive relationships depend on posttranscriptional modifications, posttranslational modifications, and protein trafficking [49, 50]. RBMS2 was noted to stabilize mRNAs in breast cancer and lung cancer [10, 21], whereas no corresponding research has been conducted in GC. We identified RBMS2 as the downstream molecule of LINC01094 and explored the function of RBMS2 in GC for the first time. Another notable mechanism identified in our study was the formation of an RNA‒DNA triplex. Many triplex-forming motifs exist in gene regulatory regions, especially the promoter region [51]. Accumulated evidence indicates that noncoding RNAs regulate target genes by participating in transcription factor recruitment and RNA‒DNA triplex formation [52, 53]. HDAC1, a member of the HDAC protein family, regulates histone modifications together with other key factors.

The axis that we proposed, a part of the complex molecular networks existing in vivo, merely provided a perspective on the exploration of LINC01094. RUNX1 was reported to inhibit CDKN1A expression through multiple pathways [54, 55], and CDK1, the known downstream gene of CDKN1A, can increase the potency of RUNX1 transactivation by phosphorylating RUNX1 at S48 or S424 [56]. These observations may suggest the existence of a regulatory feedback loop in our proposed axis.

Despite the merits of this study in providing groundbreaking insights and revealing clinical significance, its limitations should be considered. First, we chose our target gene and its downstream molecules by utilizing public databases and published studies. The sequencing data derived from the samples at our center may significantly enhance the persuasiveness and credibility of our research conclusions. While several studies have established a correlation between LINC01094 and GC, we nonetheless express regret for not having conducted sample sequencing. Second, our study involved many regulatory relationships for more comprehensive research. Sufficient rescue experiments were not performed to validate each regulatory relationship. Subsequent correlation analysis of clinical parameters and survival outcomes revealed a strong association between the expression of LINC01094 and HER2 (Fig. 1I), which we did not investigate. This correlation may offer inspiration for further studies. Moreover, recent studies have suggested that LINC01094 promotes the progression of GC through its interaction with AZGP1, which appears to contrast with our research conclusions [57]. However, I do not perceive a conflict between these two findings. In fact, it is plausible that LINC01094 facilitates GC progression via multiple pathways, thereby highlighting its potential as a therapeutic target in this malignancy. Nonetheless, further investigation is warranted to determine whether there exists an association between these two regulatory pathways. Finally, the influence of Ro 5–3335 on GC seemed to be preliminarily validated. However, much work is required before possible clinical translation.

## Conclusion

In summary, we tentatively conclude that LINC01094, regulated by the LINC01094-miR-128-3p-RUNX1 positive feedback loop, downregulates CDKN1A by interacting with RBMS2 and HDAC1 in the cytoplasm and nuclear, respectively, which in turn promotes malignant behaviors and poor prognosis in GC. These results may facilitate future exploration of a potential biomarker and pave the way for the discovery of a therapeutic target for GC.

## Supplementary Information

Additional file1 (PDF 1319 KB)

## Data Availability

No datasets were generated or analysed during the current study.

## Abbreviations

lncRNAs: Long non-coding RNAs
GC: Gastric cancer
LINC01094: Long intergenic non-protein coding RNA 1094
FISH: Fluorescence in situ hybridization
RIP: RNA immunoprecipitation
ChIP: Chromatin immunoprecipitation
CDKN1A: Cyclin dependent kinase inhibitor 1A
RBMS2: RNA binding motif single stranded interacting protein 2
HDAC1: Histone deacetylase 1
RUNX1: RUNX family transcription factor 1
nt: Nucleotides
ceRNAs: Competitive endogenous RNAs
EMT: Epithelial-to-mesenchymal transition
FBS: Fetal bovine serum
siRNAs: Small interference RNAs
qRT-PCR: Quantitative real-time reverse transcription polymerase chain reaction
ActD: Actinomycin D
CHX: Cycloheximide
DAPI: 4,6-Diamidino-2-phenylindole
TFOs: Triplex-forming oligonucleotides
IHC: Immunohistochemical
HE staining: Hematoxylin and eosin (HE) staining
DAB: Diaminobenzidine
CCK-8: Cell counting kit-8
NCBI: National Center for Biotechnology Information
OS: Overall survival
AREs: AU-rich elements
3′-UTR: 3′-Untranslated region
H3K9: H3 lysine-9
H3K9Ac: H3 lysine-9 acetylation
TrTs: Triple target site
