# Supplementary material for: LINC01094 promotes gastric cancer through dual targeting of CDKN1A by directly binding RBMS2 and HDAC1
Source: Biol Direct. 2024 Dec 25;19:137. doi: 10.1186/s13062-024-00582-y (PMC11669238; doi:10.1186/s13062-024-00582-y)
Supplement: Supplementary file 1 — Additional file1 (PDF 1319 KB) [file 13062_2024_582_MOESM1_ESM.pdf]

**siRNAs, mimics, and inhibitors**	
si-LINC01094#1	5'-CCAUGAAUGUCUUUAGUUC-3'
si-LINC01094#2	5'-CCAUAUAGGUCCUGACUAA-3'
si-RBMS2#1	5’-CCUCUGGCAUUAGAAUUAUTT-3′
si-RBMS2#2	5’-AUAAUUCUAAUGCCAGAGGTT-3 ’
si-HDAC1	5′-GCCGGUCAUGUCCAAAGUATT-3′
si-CDKN1A	5′-CCGGGTCACTGTCTTGTACCCTTGTCTCGAGACAAGGGTACAAGACAGTGACTTTTTG-3′
si-RUNX1	5′-AAGAGGUGAUGGAUCCCAGGUACUGCAGUACCUGGGAUCCAUCACCUCUU-3′
miR-128-3p Mimic	5′-UCACAGUGAACCGGUCUCUUU−3′
Mimic NC	5′-UUGUACUACACAAAAGUACUG−3′
miR-128-3p inhibitor	5′-AAAGAGACCGGUUCACUGUGA−3′
inhibitor NC	5′-CAGUACUUUUGUGUAGUACAA−3′

**Primers**		
linc01094	Forward,	5’-TGTAAAACGACGGCCAGT-3 ’
	Reverse,	5’-CAGGAAACAGCTATGACC-3 ’
linc01094 promoter	Forward,	5 ’-TGCCTCATCACTGTTGCCAT-3 ’
	Reverse,	5 ’-TATACACGCCTGGGGAAAGC-3 ’
linc01094 promoter fragment 1	Forward,	5 ’-GTAGGCCATAGCAACCCTGG-3 ’
	Reverse,	5’-ATGCACAACTCCTGCCTTCT-3 ’
linc01094 promoter fragment 2	Forward,	5’-TGAGTGAGGTGGCTTTTGAGTT-3 ’
	Reverse,	5’-CATCCAGACCTTCCTCTCTCTTC-3 ’
linc01094 promoter fragment 3	Forward,	5 ’-AGTCTGGGCTATTAACACAGGT-3 ’
	Reverse,	5’-ACACAGTGAGAACGAGAAGGG-3 ’
RBMS2	Forward,	5’-AGTTCTGACACCAGGGATGG-3 ’
	Reverse,	5′-TGCTCCTCGACTGAAACA-3′
CDKN1A	Forward,	5′-TGTCCGTCAGAACCCATGC-3′
	Reverse,	5′-AAAGTCGAAGTTCCATCGCTC-3′
CDKN1A promoter	Forward,	5′-TCCTGGCTCTAACAACATC-3′
	Reverse,	5′-TTGACAGTGGTGGTATCTC-3′
HDAC1	Forward,	5′-GACGGCATTGACGACGAATC-3′
	Reverse,	5′-TGAAGCAACCTAACCGGTCC-3′
RUNX1	Forward,	5′-GATGGCACTCTGGTCACCG-3′
	Reverse,	5′-GCCGCTCGGAAAAGGACAA-3′
miR-128-3p	Forward,	5’-CGGGCTCACAGTGAACCGG-3 ’
	Reverse,	5’-CAGCCACAAAAGAGCACAAT-3 ’
GAPDH	Forward,	5’-AATGGATTTGGACGCATTGGT-3 ’
	Reverse,	5′-TTTGCACTGGTACGTGTTGAT-3′
U6	Forward,	5′-CTCGCTTCGGCAGCACA-3′
	Reverse,	5′-AACGCTTCACGAATTTGCGT-3′

18S	Forward,	5′-AACTTAAAGRAATTGACGGA-3′
	Reverse,	5′-TCCGTCAATTYCTTTAAGTT-3′
**Antibodies**		
RBMS2	67395-1-Ig	Proteintech
HDAC1	10197-1-AP	Proteintech
CDKN1A	10355-1-AP	Proteintech
E- cadherin	20874-1-AP	Proteintech
N - cadherin	22018-1-AP	Proteintech
Vimentin	10366-1-AP	Proteintech
GAPDH	60004-1-Ig	Proteintech
Ki67	28074-1-AP	Proteintech
RUNX1	ab272456	Abcam
CDK4	23972	Cell Signaling Technology
CDK6	13331	Cell Signaling Technology
Cyclin D1	55506	Cell Signaling Technology
Cyclin E1	20808	Cell Signaling Technology
RELN	ab78540	Abcam
TUB	17928-1-AP	Proteintech
**Websites**		
NCBI	https://www.ncbi.nlm.nih.gov/	
TCGA	https://www.cancer.gov/ccg/research/genome-sequencing/tcga	
GEO	https://www.ncbi.nlm.nih.gov/geo/	
lncATLAS	https://lncatlas.crg.eu/	
Kaplan-Meier Plotter	http://kmplot.com/analysis/index.php?p=service&cancer=pancancer_rnaseq	
GSEA	https://www.gsea-msigdb.org/gsea/index.jsp	
UniProt	https://www.uniprot.org/	
CatRAPID	http://service.tartaglialab.com	
LongTarget	http://www.gaemons.net/LongTarget	
JASPAR	https://jaspar.genereg.net/	
LncSNP2 .0	http://bioinfo.life.hust.edu.cn/lncRNASNP/#!/	
AnnoLnc2	http://annolnc.gao-lab.org/	
TargetScan	https://www.targetscan.org/vert_80/	
miRWalk	http://mirwalk.umm.uni-heidelberg.de/	
miRTarBase	https:// mirtarbase.cuhk.edu.cn/~ miRTarBase/miRTarBase_2022/ php/index.php	
miRmap	https://mirmap.ezlab.org/app/	
DIANA	http://diana.imis.athena-innovation.gr/DianaTools/index.php	

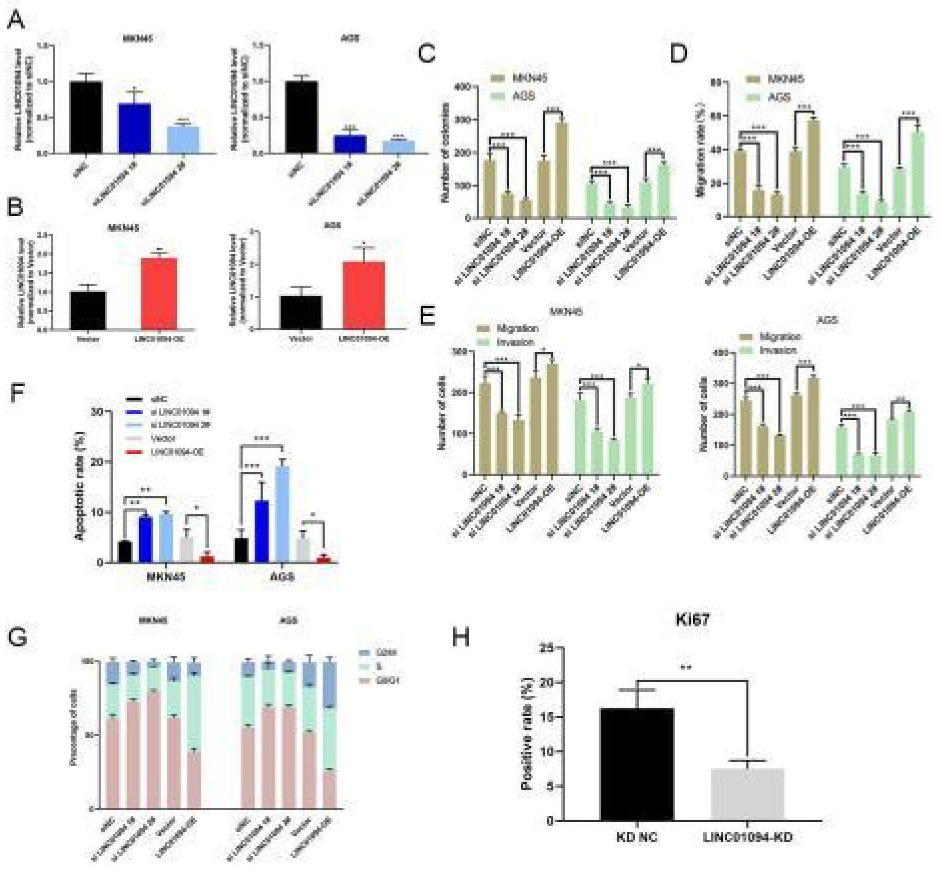

Figure S1

Notes: (A-B) The knockdown **(A)** and overexpression **(B)** efficiencies of LINC01094 was validated by qRT-PCR in MKN45 and AGS cells. **(C-H)** The statistical analysis of colony formation assays **(C)**, wound healing assays **(D)**, transwell assays **(E)**, apoptotic rate **(F)**, cell cycle **(G)**, and IHC **(H)** in **Figure 2** and **Figure 3**.

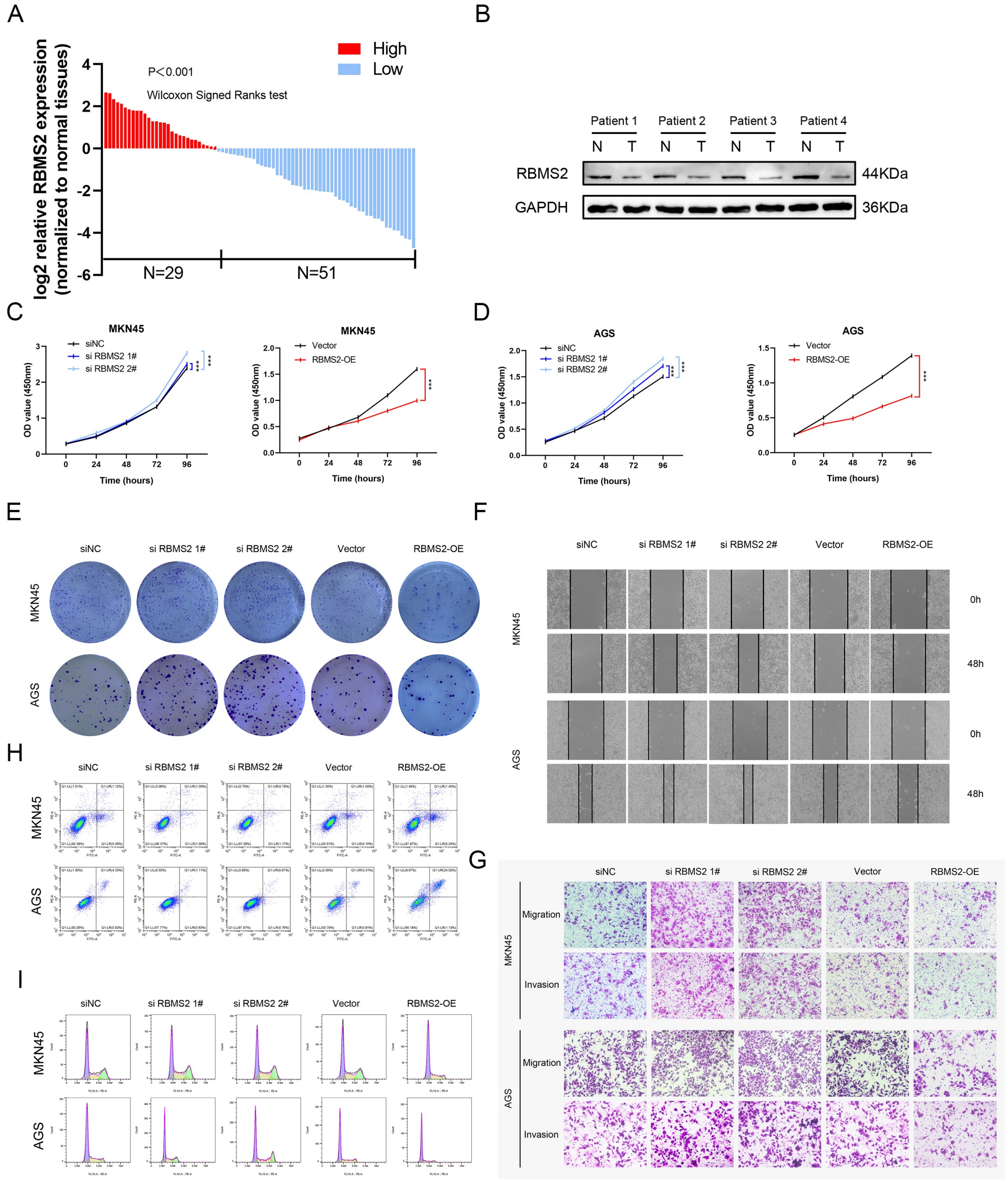

Figure S2 RBMS2 is lowly expressed in GC and inhibits the malignant behaviours of GC cells in vitro.

Notes: **(A-B)** qRT-PCR **(A)** and western blot **(B)** showed that RBMS2 was .downregulated in GC tissues. **(C-I)** RBMS2 inhibited cell proliferation **(C-E)**, migration and invasion **(F-G)**, but promoted apoptosis **(H)** and G1 to S transition **(I)** in GC cells.

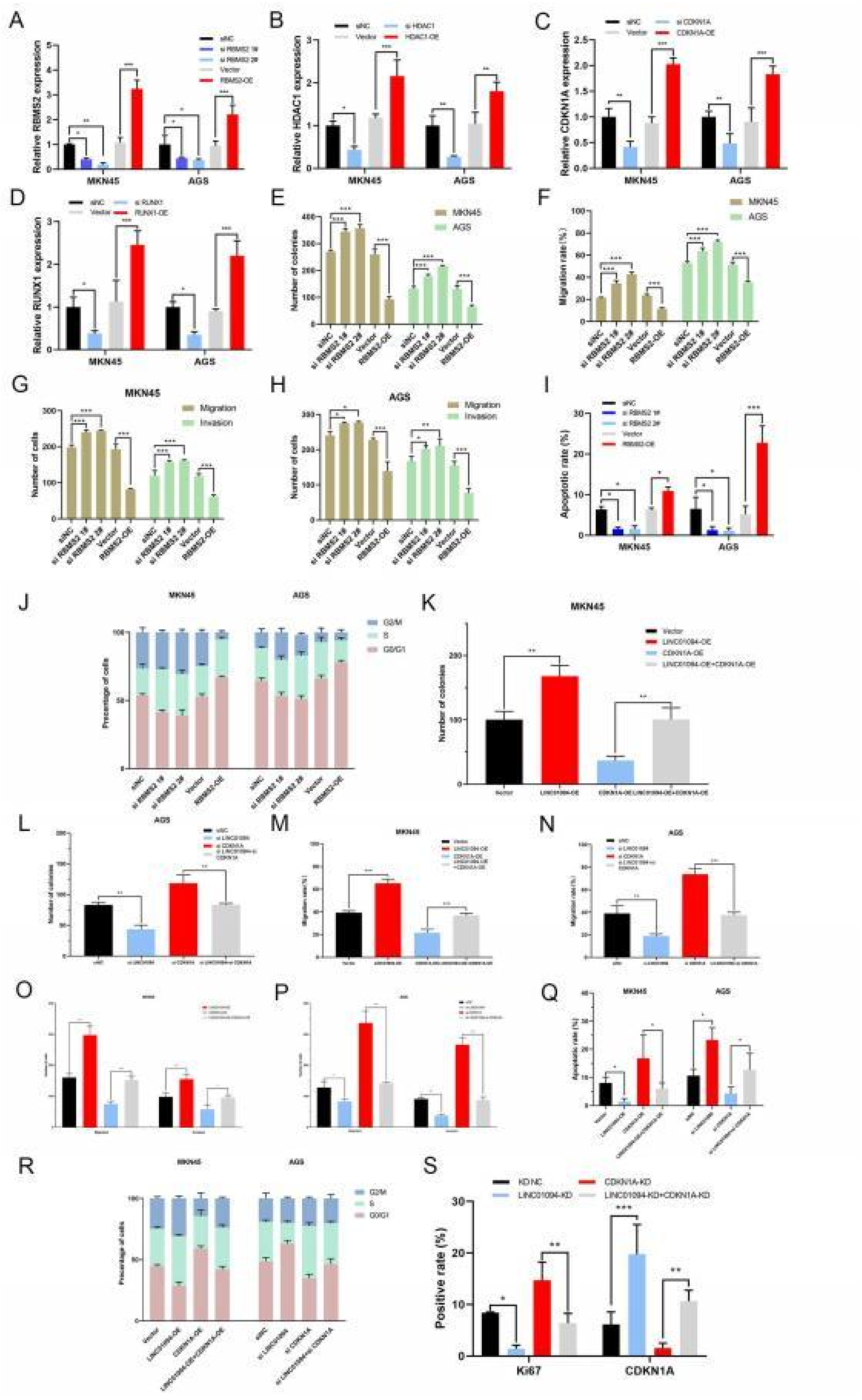

Figure S3

Notes: (A-D) The knockdown and overexpression efficiencies of RBMS2 **(A)**, CDKN1A **(B)**, HDAC1 **(C)**, and RUNX1 **(D)** were validated by qRT-PCR. **(E-J)** The statistical analysis of colony formation assays **(E)**, wound healing assays **(F)**, transwell assays **(G-H)**, apoptotic rate **(I)**, and cell cycle **(J)** in **Figure S2**. **(K-S)** The statistical analysis of colony formation assays **(K-L)**, wound healing assays **(M-N)**, transwell assays **(O-P)**, apoptotic rate **(Q)**, cell cycle **(R)**, and IHC **(S)** in **Figure 7**.

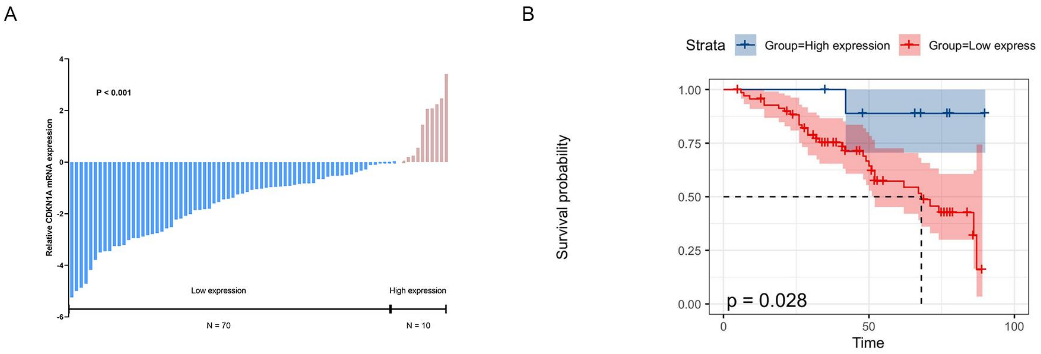

Figure S4 CDKN1A is downregulated in GC and correlates the prognosis of GC patients.

Notes: (A) qRT-PCR revealed that CDKN1A is downregulated in tumor tissues compared to the corresponding normal tissues. **(B)** The Kaplan–Meier showed that patients exhibiting lower level of CDKN1A have worse prognosis.

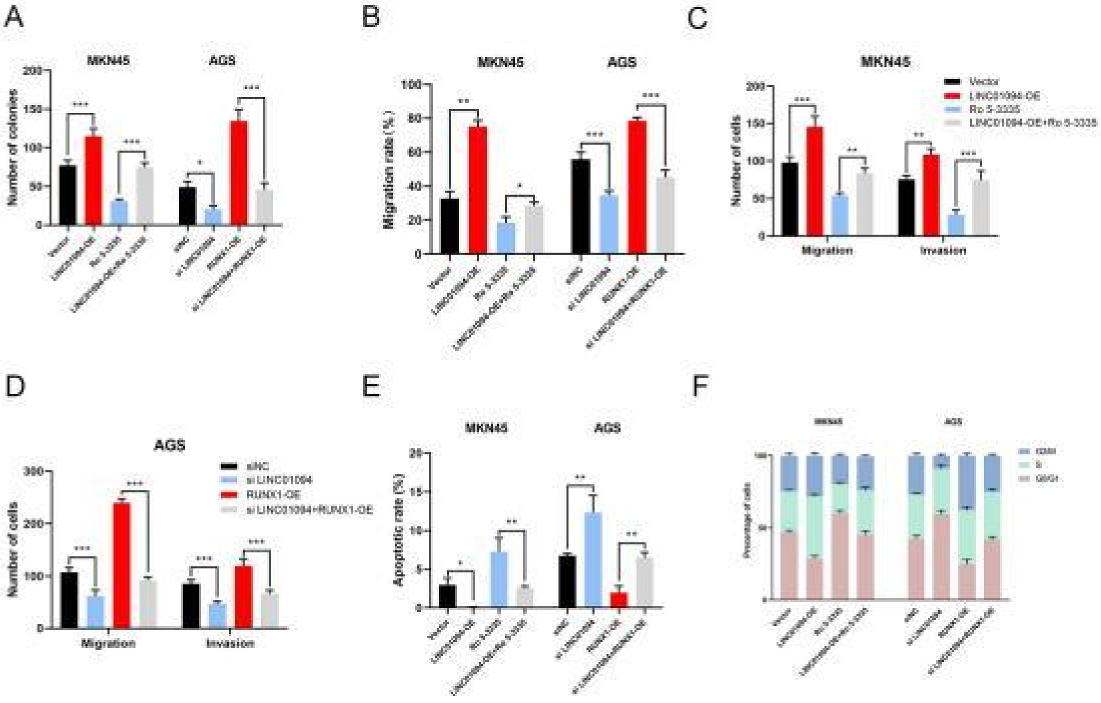

Figure S5

Notes: (A-F) The statistical analysis of colony formation assays **(A)**, wound healing assays **(B)**, transwell assays **(C-D)**, apoptotic rate **(E)**, and cell cycle **(F)** in **Figure 9**.
